# Consequences of the Pandemic on Mental Health of Healthcare Workers in the NHS

**DOI:** 10.3390/bs14121208

**Published:** 2024-12-17

**Authors:** Arjun Vyas, Nantapong Butakhieo, Lina Vyas

**Affiliations:** 1 James Cook University Hospital, South Tees NHS Foundation Trust, Middlesbrough TS4 3BW, UK; arjun.vyas3@nhs.net; 2 Department of Social Sciences and Policy Studies, The Education University of Hong Kong, Hong Kong SAR, China; nbteddy@s.eduhk.hk

**Keywords:** healthcare worker, turnover intention, frustration at work, NHS, UK

## Abstract

In recent years, the public health system of the United Kingdom, the National Healthcare System (NHS), has encountered difficulties that have been acknowledged in research studies and public policy discussions, such as resignations and staff shortages. During the COVID-19 pandemic, NHS healthcare workers were confronted with demanding circumstances, exacerbating the constraints of an already struggling system. With this, the authors of this paper aim to better understand the relationships between frustration at work, fear of infection, working hours, and the turnover intention of healthcare workers during the pandemic. This study employed a mixed-methods research approach, as a questionnaire survey was conducted along with an online self-administered interview questionnaire. Using mediation and moderated mediation analyses, it was found that the indirect effect of frustration at work through fear of infection on turnover intention was positively significant. Working hours moderated the mediation effect of fear of infection on the relationship between frustration at work and turnover intention. Surprisingly, the conditional indirect effect of frustration at work on turnover intention through fear of infection was the strongest among those with short working hours. This evidence was supplemented with qualitative results that enhance the understanding of why healthcare workers want to leave the system and the actions that can be taken on the organisational and policy fronts to address this issue.

## 1. Introduction

The healthcare sector is considered one of the most prestigious and well-respected industries. With the positive reputation of being employed in the industry, healthcare workers (HCWs) face challenges on both occupational and personal fronts. Research indicates that the healthcare industry tends to be associated with stress and work frustration issues due to multiple factors: it is highly competitive; has demanding hours; and is a high-stake working environment [1]. These issues have been found to have an impact on turnover intention, especially among HCWs [2]. The obligation and responsibility towards their profession motivated HCWs to persist in their roles despite the difficulties posed by the pandemic [3]. This often results in them sacrificing their own personal time to meet job demands. This was particularly exacerbated during the pandemic.

The COVID-19 pandemic presented new challenges to the already tough situation faced by HCWs globally. From the fear of being infected to not seeing friends and family, they faced immense difficulties. HCWs were required to work long shifts, and the constant fear of infection added to their frustration at work and stress levels [4]. The staff of the National Healthcare System (NHS) in the UK are no exception. The NHS is one of the largest employers in the world, with over 1.5 million employees, covering primary care (e.g., general practitioners) to specialist hospital care [5]. The pandemic significantly impacted the UK’s healthcare delivery, revealing issues within the NHS, such as unpreparedness [6], under-resourced facilities [7], and staff shortages [8]. Due to the high transmission rate of COVID-19 and its severe symptoms, hospitals—which were at the forefront—faced peak demands that frequently surpassed their capacity to provide the required care [9].

With hospitals understaffed and the personnel at risk of infection, HCWs were under heightened stress and pressure. This has resulted in long-term mental health difficulties. The British Medical Association [10,11] revealed that about 45% of doctors suffered from depression, anxiety, stress, or other mental health conditions relating to, or made worse by, the COVID-19 crisis. Furthermore, 59% of doctors expressed that their level of fatigue or exhaustion had been higher than normal during the pandemic. Likewise, a survey conducted by Nursing Times in 2023 found that 40% of nurses reported that their mental health was “worse” or “much worse” than it was in 2020 or 2021 [12]. 

Previous studies have shown that the NHS workforce has long been affected by high turnover rates [13,14]. The fear of COVID-19 infection and frustration at work caused by the pandemic may have further exacerbated this issue [15]. Likewise, they have discussed factors contributing to HCWs’ turnover intention prior to the pandemic, such as long working hours [16]. However, it remains unclear whether these factors became more or less significant in influencing turnover intention among HCWs during the pandemic [17]. Previous studies in the literature have mainly focused on the impact of job stress, burnout, and fear of infection during the pandemic [18,19]. However, there is a limited understanding of the relationship between frustration at work, fear of COVID infection, working hours, and turnover intention among HCWs, particularly in the UK and during unique times such as the pandemic.

The present study addresses the impact of the pandemic on HCWs in the UK healthcare system. The significance of this study lies in its exploration of the increased turnover intention among HCWs, which poses risks to an already burdened system. This paper presents a holistic overview of the situation via collaborative work between academicians and healthcare practitioners. This study also holds significance, as the authors explore the mediating factor of fear of infection on the impact of frustration at work on the relatively high turnover rate of HCWs in the UK. It is vital to recognise the impact of frustration at work and fear of infection on the turnover intention of HCWs, as a strong healthcare system is considered the backbone of any nation. With this, this study aims to assess the relationship between frustration at work, fear of infection, working hours, and turnover intention among HCWs in the UK during the pandemic. In addition, this study seeks to contribute to the development of strategies to sustain and support the healthcare workforce beyond the pandemic, supplemented with qualitative results that enhance the understanding of why HCWs want to leave the system and the actions that can be taken on the organisational and policy fronts to address this issue.

## 2. The Four Waves of Pandemic in the UK

As the UK experienced the spread of COVID-19, the government response varied from introducing lockdowns and social distancing measures to mass vaccination campaigns [20]. This summarising of the pandemic’s timeline could provide a holistic view of the medical pressure and the impact of the pandemic on the health sector. There were four apparent pandemic waves in the UK, which are contextualised in Table 1.

## 3. Literature Review

### 3.1. Turnover Intention in the Health Sector

Turnover intention refers to the cognitive decision-making process of considering whether to quit one’s job, and it is generally accepted as a predictor of actual voluntary job resignation [26]. According to Hayes [27], turnover intention in the healthcare sector can be described as the inclination of HCWs to switch occupations to another field of employment or change positions in the same industry. Derycke et al. [28] investigated the impact of personal and occupational factors on the turnover intention of HCWs and found that there was a strong correlation between perceived work ability and turnover intention. Employees who were more confident in their abilities were less likely to display the intention to resign from their positions. HCWs with a higher skillset were less likely to have a high turnover intention.

With the unprecedented situation caused by the pandemic, further investigation has been conducted to discuss the causes of HCWs’ turnover intention. A study by Hou et al. [29] found that various factors were connected to the turnover intention of HCWs in China and that they were complex and varied. The study’s findings indicated that the likelihood of turnover intention is 1.78 times higher in hospitals with limited resources compared to those with ample resources. Furthermore, an association was found between turnover intention and both depression and a low level of social support. The study pointed to the early detection of COVID-19 among patients as a means to improve the turnover intention of the workers. Similarly, Poon, Lin, Griffiths, Yong, Seah, and Liaw [17] examined the turnover intention of HCWs amid the pandemic and identified multiple influencing factors, including individual, interpersonal, job-related, and organisational determinants; many of these were known factors prior to the pandemic, including psychological responses to stress, socio-demographic characteristics, adverse working conditions, and organisational support, while a fear of COVID-19 exposure was unique and specific to the pandemic [17]. With the above literature, it can be seen that various factors impact the turnover intention of HCWs and that the issue was amplified by the pandemic.

### 3.2. Working Hours and Turnover Intention

Long working hours can refer to the duration of work that significantly exceeds the standard or average working time; long working hours have a negative impact on workers’ health [30]. HCWs have long faced challenges relating to long working hours; those employed by the UK’s NHS worked excessive hours even prior to the pandemic, with many working more than 50 h per week [31]. Likewise, several studies have been conducted to explore the impact of working hours on the turnover intention of HCWs during the pandemic [29,32,33]. HCWs worked under the constant fear of infection, and staff shortages aggravated the circumstances that they faced. As infections among HCWs rose, hospitals became increasingly understaffed. With this, HCWs were required to work longer hours to meet the demand for care services [34,35].

Previous studies found that longer working hours were associated with a higher turnover intention, both before [36,37] and during the pandemic [29,32,33]. Galanis et al. [38] found that, in the situation of a staff shortage, turnover intention was exacerbated among nurses who did not receive the right organisational support as compared to those who experienced more organisational support, which was found to reduce the turnover intention. This organisational support refers to the organisation caring about the well-being of its employees and valuing their contributions [39]. Furthermore, the response strategies adopted during the pandemic, such as high pandemic preparedness and response resources, e.g., PPE and other protective gear for frontliners, had an impact on the turnover intention of HCWs, suggesting that the work environment plays a significant role in their intention to leave their positions [17]. Moreover, a study by Buchan et al. [40] explored the relationship between turnover intention and the nursing staff shortage experienced during the pandemic. It was found that HCWs who endured significant stress in the workplace were at a greater risk of leaving their position. This supports the argument that the work environment, linked to long working hours, has an impact on the turnover intention of HCWs.

### 3.3. Frustration at Work, Fear of Infection, and Turnover Intention

Studies have been conducted to examine the factors contributing to the turnover intention of HCWs in various countries [41,42]. Employees’ frustration at work is the psychological process experienced by employees when they are constantly frustrated during their work [43,44]. Organisations can cause frustration at work due to, for example, unsuitable work arrangements and inappropriate use of resources, and frustration at work is the subjective feelings of an individual resulting from work events [45]. It was found that a shortage of workforce, low payment, and long working hours can be considered as frustration at work-predicting factors [45]. Van Dick et al. [46] asserts that a heavy workload among HCWs, often caused by a shortage of staff, reduces the time available for family responsibilities and leisure activities, consequently increasing their risk of stress and work frustration. Strong frustration at work experienced by HCWs tends to lead them to have a strong desire to leave their jobs [45].

A few studies have examined the associations between frustration at work and turnover intention among HCWs. For example, McHugh, Kutney-Lee, Cimiotti, Sloane, and Aiken [44] established that job frustration, as a negative emotional response, is positively associated with employee emotional exhaustion and an excessive workload, which in turn contributes to an increased intention to leave the job. Research has shown that various work characteristics—such as heavy workloads, urgency, responsibility, task complexity, and interpersonal interactions with patients and colleagues—can create significant stressors for nurses. This pressure can result in feelings of anger and negative experiences, ultimately leading to frustration with their work, which may subsequently evolve into intentions to resign [44,45,47]. The heightened stress experienced by healthcare workers during the pandemic has intensified their frustration at work, highlighting the need to explore the impact of frustration at work on turnover intentions among HCWs.

Previous research states that work-related stress arises when an individual perceives that their job’s demands are strenuous or surpass their capacity to cope [48]. Although previous research focuses on the association between other sources of frustration at work and work-related stress (such as over workload and job insecurity) [49,50], the present study focuses on the fear of COVID infection experienced by HCWs as the primary source of stress, referring to the stress and worry of working under high pressure due to the circumstances caused by the pandemic. Likewise, Nestor, O’ Tuathaigh, and O’ Brien [34] and Wang et al. [51] indicated that the main sources of stress among HCWs included a fear of contracting COVID-19 or transmitting it to friends/family. Walker and Tolentino [52] also pointed out that the unstable hospital COVID-19-related policies and protocols along with the switching of the roles of responsibilities of HCWs added to their stress. In addition to occupational factors, the constant fear of infection contributed to the stress of HCWs.

In the UK, after the peak of COVID-19 in April 2020, a survey conducted among 2773 HCWs revealed that poor mental well-being was prevalent during the COVID-19 response, which resulted in high levels of anxiety, depression, post-traumatic stress disorder, and stress [8]. Stressful conditions and fear of infection were exacerbated by insufficient information, pressure to work without PPE, more than 20% of team members being off sick, and the perception that not enough was being done to mitigate risk. Further, Choudhury et al. [53] reported that HCWs showed signs of mild depression, heightened stress, and anxiety during the pandemic, which implied a significant risk of staff experiencing frustration at work under the said conditions. A study by Karimi et al. [54] on the turnover intention and actual turnover among nurses during the pandemic revealed that there was a significant correlation between stress, anxiety, and a fear of COVID-19 infection, which, in turn, increased the intention to leave among nurses. 

**Figure 1:**
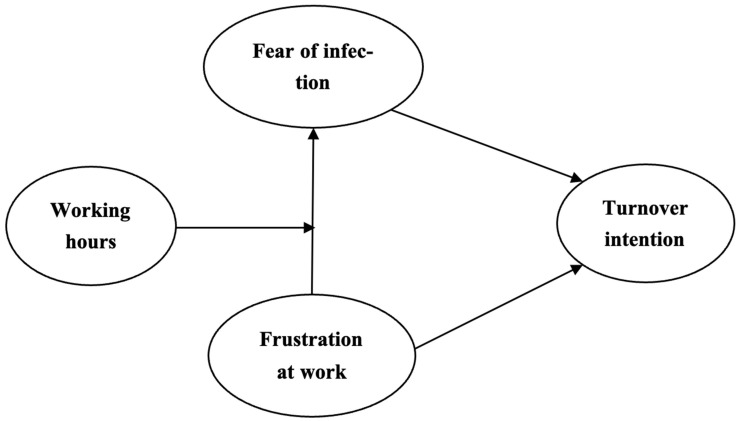
Hypothesised relations in mediation and moderated mediation models.

It can be seen in the previous literature that the unprecedented situation caused by the pandemic put stress and frustration on the HCWs. As mentioned above, HCWs were under frustration at work and fear of infection, which was impacted by various personal and organisational factors. This study investigates whether the circumstances, fear of COVID infection, acted as a mediator and influenced the impact of frustration at work on intention to leave among HCWs. The NHS’s HCWs were already unhappy with their work environment [55], were unsatisfied with the protection given by the government, and lacked trust in the government’s protocols to ensure their safety during the pandemic [56], which probably exacerbated their fear of infection [57]. This combination of frustration and fear may have led to an increased intention among HCWs to leave their jobs. Moreover, this study aimed to better understand whether working hours moderated the relationship between frustration at work and turnover intention through the mediator of fear of infection. This will help to enhance the understanding of how the relationship between frustration at work, fear of infection, and turnover intention develops with different numbers of working hours. Further to testing the relationship between the aforementioned variables, this study also reveals the reasons for HCWs’ turnover intention by the supplementation of the qualitative results. A conceptual diagram of the hypothesised relations in the mediation and moderated mediation models is shown in Figure 1. This study thus tested the following hypotheses:

**Hypothesis** **1.** *Fear of infection mediates the relationship between frustration at work and turnover intention.*

**Hypothesis** **2.** *Working hours moderate the indirect effect of frustration at work through fear of infection on turnover intention.*

## 4. Methods

This study was conducted to test the relationship between frustration at work and turnover intention among HCWs in the UK during the pandemic using a mixed-methods research approach. The HCWs in this study include nurses, doctors, therapists, and pharmacists who are employed by the NHS. The authors conducted a questionnaire survey followed by an online self-administered interview questionnaire. With this approach, the data can be corroborated, allowing for a more comprehensive understanding of the research topic. This, in turn, validates the research findings [58]. Ethical approval for this study was obtained from the Human Research Ethics Committee (HREC) of the authors’ affiliated university.

Using snowball and network sampling, quantitative data were collected from the online questionnaire from April to August 2022, which surveyed individual HCWs working in the UK’s NHS during the COVID-19 pandemic. With this sampling method, data can be collected based on referrals, allowing for a more cost-effective method of collecting data. In response to travel and geographic restrictions during the pandemic [59], the online questionnaire was distributed to HCWs via email and social media (e.g., WhatsApp and Facebook). The online questionnaire was created using Qualtrics web-based survey software. It took approximately 7 to 9 min to complete the survey questionnaire, and it received a total of 145 responses, of which 119 were determined to be valid. Validity is contingent upon completeness, meaning that invalid responses resulted from incomplete questionnaires. Participation in this study was voluntary, and all collected data were kept confidential.

The questionnaire primarily utilised “ranking” questions, which were adapted from Caillier [60], Salas-Vallina et al. [61], Campbell and Popescu [62], and Bae et al. [63], focusing on the respondents’ attitudes towards turnover intention and their frustration at work, as well as fear of infection. Individual demographic information included age, gender, and profession category. Other job-related information collected included the place of work, monthly salary, working years, working schedule, and working hours. The HCWs were asked to rate their opinions on questions related to each variable; a 5-point scale was used for frustration at work and turnover intention, where “1 = Strongly disagree” and “5 = Strongly agree”, and a 4-point scale was used for fear of infection, where “1 = Do not worry” and “4 = Worry a lot”. For instance, frustration at work was measured by the question “I am often frustrated at work, and this feeling won’t go away”. Similarly, turnover intention was measured by “I will likely actively look for a new job within the next two years.” Fear of infection was measured by “Do you worry about whether you have been infected with COVID-19?” The working hours variable refers to the average number of hours worked per week during the pandemic, excluding meal breaks. The collected data were analysed in a statistical analysis using IBM SPSS version 29. 

Qualitative data were collected through an online qualitative survey [61]. The participants were selected from the pool of questionnaire respondents. The authors developed interview questions on the basis of the quantitative results. In order to encourage the healthcare participants, who usually had busy schedules and heavy workloads, the researchers opted to collect qualitative data using an online self-administered interview questionnaire through the Qualtrics survey platform. This data collection approach helped to overcome the workforce and time constraints, as the HCWs could answer the questions at a time convenient to and suitable for their schedule. While traditional qualitative data collection relies heavily on qualitative interviews, prior research indicates that qualitative surveys can also yield valuable qualitative data, as highlighted in a study by Braun et al. [64]. The interview questions comprised a combination of open, open-ended, probing, and leading questions. Six interview questions were included in the online self-administered interview questionnaire. For instance, one question asked, “What types of job-related stress have you experienced, and how did they affect you? To what extent are these job-related stresses connected to the pandemic?” Another question inquired, “Are you planning to look for a new job within the next two years? Why or why not?”. The interview was expected to take approximately 15 min to complete. A total of 18 valid responses were received. Creswell and Creswell [58] and Creswell and Báez [65] suggest analysing qualitative raw data using a thematic coding process. In this process, each text is coded to identify key themes. Through coding, various codes are gathered to support the themes. Braun, Clarke, Boulton, Davey, and McEvoy [64] suggest that coding is one of the most productive ways to analyse qualitative survey data. Likewise, content analysis is a method used to examine text data, allowing researchers to analyse and interpret their meaning [66,67]. Thus, this study analysed the qualitative data utilising thematic coding and a content analysis to supplement the quantitative results.

## 5. Findings

### 5.1. Quantitative Findings

A total of 119 healthcare participants completed the survey. Of the total sample, 75 were female, and the majority were doctors. Table 2 illustrates the sample characteristics.

Using a correlation analysis (see Table 3), no significant direct relationship was found between turnover intention and frustration at work among the HCWs (*p* > 0.10). However, the relationships between fear of infection and turnover intention (*p* < 0.01) and between fear of infection and frustration at work (*p* < 0.05) were both significant.

For Hypothesis 1, this study examined whether fear of infection mediated the relationship between frustration at work and turnover intention among HCWs. The authors conducted a bootstrapping analysis (with 5000 bootstrapping samples) in SPSS to test for mediation using model 4 of the PROCESS Macro developed by Hayes (2017) [27]. The results in Table 4 show that the total effect of frustration at work on turnover intention was positively significant (*B* = 0.23, *p* < 0.01). Frustration at work was significantly positively associated with fear of infection (*B* = 0.32, *p* < 0.01), but the direct effect of frustration at work on turnover intention was not statistically significant. Fear of infection was significantly positively associated with turnover intention (*B* = 0.25, *p* < 0.01). The indirect effect of frustration at work through fear of infection on turnover intention was positively significant ((*B* = 0.08), with a 95% percentile bootstrap confidence interval (BPCI) = [0.01, 0.19]), which indicates full mediation. Thus, Hypothesis 1 is supported. The results suggest that HCWs with higher frustration at work levels tended to have more fear of infection and, hence, a higher turnover intention.

For Hypothesis 2, the hypothesised moderated mediation model was tested using model 7 of the PROCESS Macro with 5000 bootstrapping samples; additionally, the model that included the moderator of working hours in the effect of the mediation path was tested. In the moderated mediation model, the interaction between frustration at work’s direct effect on fear of infection was seen to be statistically significant (*B* = 0.30, *p* < 0.001, 95%CI = 0.139, 0.468). Table 5 indicates that working hours moderated the indirect effect of frustration at work on turnover intention through fear of infection. The overall moderated mediation model was supported with the index of moderated mediation = −0.036 (95%CI = −0.092, −0.002). The conditional indirect effect was the strongest among those with short working hours (1 SD below the mean of working hours; effect = 0.13, SE = 0.07, 95%CI = 0.017, 0.287) and the weakest among those with long working hours (1 SD above the mean of working hours; effect = 0.02, SE = 0.03, 95%CI = −0.029, 0.097).

Additionally, the participants from the online qualitative survey were asked to read the statements in Table 6 and rate them with a number from 0 to 3 to indicate how much they applied to them over the past week, with 3 indicating that they applied to them very much or most of the time. The results show that most respondents had more than one of the six negative feelings listed above. For most respondents, negative feelings were mild, while a few respondents felt severe negative feelings and experienced them most of the time. 

### 5.2. Qualitative Findings

Regarding the qualitative interviews, the pool comprised 18 physicians (8 females, 9 males, and 1 respondent who preferred not to say). The thematic coding and content analysis results revealed four main themes, namely, work-related negative feelings, causes of stress, turnover intention, and retention, as discussed below:

(1) Work-related negative feelings: The results indicate that the HCWs experienced negative feelings during their work amid the pandemic. Most reported that their negative emotions were related to the six specific statements listed in Table 6, all of which pertained to work-related issues. In particular, younger doctors expressed a greater extent of the negative impact of work on their personal lives. Many of these negative feelings stemmed from an increased workload due to staff shortages. Additionally, younger healthcare workers often felt that their contributions were undervalued and not adequately recognised. These work-related challenges can contribute to heightened stress levels among HCWs. Below are excerpts taken from the interview responses of selected respondents.

When things are tough for an extended amount of time, i.e., understaffing, repeated emergencies, difficult consultants, etc., it can wear you down and make you not value yourself.

(Doctor Number 12, Male, 26 years old)

Mostly work related, particularly with regards to question 6. The training is becoming more competitive in the UK, and with sub-inflationary pay rises for decades it feels as though I could have a less stressful time and be paid better in other professions or overseas.

(Doctor Number 11, Male, 25 years old)

The work carries on till late evening- mainly paper work.

(Doctor Number 4, Female, 56 years old)

Can work extra hard due to insufficient staffing and unmanageable caseload, however, get little to no recognition for this.

(Doctor Number 13, Male, 29 years old)

Not valued as doctor at work. Not appreciated by team, senior or patients

(Doctor Number 17, Male, 27 years old)

(2) Causes of stress: All the interview participants indicated that their jobs are stressful to varying degrees, with different underlying causes. The respondents cited heavy workloads and a lack of resources as the main sources of stress. They felt a lack of organisational support in terms of manpower, technology, and funds. Senior doctors said that they needed to balance patient care with research programmes. Young doctors felt pressure in terms of working hours and human resource allocation. Most respondents cited the increased number of patients caused by the pandemic as stressful. Some mentioned that the stress had always been there and that it had nothing to do with the pandemic. These results are supported by the following excerpts from selected respondents’ answers:

Research projects behind deadlines and less funding for staff due to pandemic.

(Doctor Number 1, Male, 53 years old)

Long hours, responsibility for over 200 patients on night shifts, unsafe staffing levels, inability to escalate to seniors due to lack of staffing. I imagine these are all hangover effects post-pandemic.

(Doctor Number 14, Female, 25 years old)

Time pressures, stretched resources. Still feeling impact of pandemic (patients presenting later, etc.).

(Doctor Number 7, Female, 44 years old)

Poor sleep pattern and routine caused by many on-call/overnight shifts. Patients also present with a worsening of their chronic disease due to lack of GP/hospital contact over the pandemic which makes their clinical management more complicated and their prognosis poorer.

(Doctor Number 13, Male, 29 years old)

Pressure and lack of support and positive feedback.

(Doctor Number 8, Male, 71 years old)

Dealing with things beyond my competency due to lack of senior support, dealing with difficult patients.

(Doctor Number 17, Female, 26 years old)

The system is overloaded, so I am having to cover more patients at a point where it is burning me out. The pandemic made the job more stressful, but I believe it only uncovered issues that were already present in the system.

(Doctor Number 11, Male, 25 years old)

(3) Turnover intention: The qualitative results also show that almost two-thirds of the interview respondents expressed their intention to leave their current positions. They indicated that they would look for new jobs within the next two years. Most of them were likely to seek positions that offered higher salaries and career growth opportunities, including training access. Some example answers from the respondents are as follows:

I am looking to see if I can leave the country and train overseas, where training is better, job is better paid, and the system is not so overloaded.

(Doctor Number 11, Male, 25 years old)

Probably (looking for a new job), (because of) political situation in the UK including Brexit.

(Doctor Number 1, Male, 53 years old)

Maybe looking for more portfolio options (i.e., part time roles outside of clinical medicine).

(Doctor Number 7, Female, 44 years old)

(I) need a change.

(Doctor Number 8, Male, 71 years old)

(4) Retention: Overall, the HCWs showed a substantial likelihood of remaining in their positions and had a positive attitude towards their jobs. Several HCWs believed that being a doctor is a stable and well-paying profession. Additionally, senior HCWs, particularly older doctors nearing retirement, were the most likely to continue in their roles. In contrast, younger HCWs focused on career advancement; for instance, many young and middle-aged doctors plan to participate in training programmes to improve their professional level. Some doctors are aiming to change their working environment, but they have not given up their profession. These results are supported by the following excerpts from selected respondents’ answers:

I have a very senior position which I won’t give up for no good reason.

(Doctor Number 6, 53 years old)

I won’t be looking for a new job, as it’s a secure job with good pay.

(Doctor Number 18, Male, 27 years old)

I will be on a 2-year training programme from August.

(Doctor Number 13, Male, 29 years old)

I have a job on a training program for the next three years which is a new job compared to the one I have currently.

(Doctor Number 14, Female, 25 years old)

I have recently taken a year out of training.

(Doctor Number 16, Female, 26 years old)

## 6. Discussion

This study explored the relationship between frustration at work, fear of infection, working hours, and the turnover intention of NHS HCWs. The following hypotheses were proposed: fear of infection mediates the relationship between frustration at work and turnover intention (Hypothesis 1), and working hours moderate the indirect effect of frustration at work through fear of infection on turnover intention (Hypothesis 2). This study’s results support both hypotheses.

Regarding the first hypothesis, the results show no direct effect of frustration at work on turnover intention. There were statistically significant associations between frustration at work and fear of infection and between fear of infection and turnover intention among HCWs. Additionally, the indirect effect of frustration at work on turnover intention through fear of infection was statistically significant. This indicates a causational model, where frustration at work results in increased fear of infection, which leads to an increased turnover intention. This is further contextualised through the qualitative responses gathered, in which participants tended to have work-related negative feelings during the pandemic. This study’s findings are consistent with a systematic review by Poon, Lin, Griffiths, Yong, Seah, and Liaw [17], who found that fear of infection could increase the turnover intention of HCWs. Likewise, it was found that fear of COVID-19 could increase turnover intention among nurses [15]. However, the results of this study are not in line with those of Chang, Lee, and Wang [45], who found that frustration at work among nurses influenced their turnover intention. Another study also found that turnover intention was directly affected by work frustration [68]. The fear of COVID-19 infection and its associated circumstances has been recognised as a significant psychological stressor [69]. Thus, the fear of infection might be viewed as a form of stress for HCWs who are directly confronted with the disease. Furthermore, previous research indicates that psychological stress positively correlates with workers’ turnover intention [36,70]. This fear could lead to an increased HCWs’ turnover intention, as demonstrated by the results of this study.

Through the qualitative responses, no single cause of the increased stress levels in doctors could be determined; although such issues may certainly have existed pre-pandemic, the overwhelming patient load caused by the pandemic exacerbated them, and it could increase the level of frustration at work. This can be seen in the responses of both Doctors 13 and 14 in the Qualitative Results Section, who discuss how patients’ pre-existing diseases were further exacerbated by the pandemic, taking attention away from these patients’ ongoing care. With this, it can be said that the stress triggered by the COVID-19 pandemic can also be classified as “work-related stress” experienced by the HCWs. The heavy workload and lack of support and resources from the organisation, especially during the four waves of the COVID-19 pandemic (see Table 1) in the UK, may have increased HCWs’ stress levels and frustration at work may have been exacerbated. It was also observed that the HCWs tended to look for new job opportunities or participate in training programmes to enhance their professional skills and change their work environment, as indicated by the qualitative findings. In contrast, the qualitative results indicated that career advancement was considered a key retention factor for younger HCWs.

Regarding the second hypothesis, the quantitative results showed that working hours moderated the mediation effect of fear of infection on the association between frustration at work and turnover intention. From the survey conducted, it was concluded that frustration at work was positively associated with fear of infection, which, in turn, led to a higher turnover intention among the HCWs. Surprisingly, the moderated mediation model revealed that the association between frustration at work and fear of infection was the strongest among those with short working hours. As the fear of infection derived from COVID-19 and its surrounding circumstances, this may imply that HCWs who had shorter working hours during the COVID-19 pandemic, especially during the four waves of the COVID-19 pandemic in the UK, had increased worries and a fear of COVID-19 infection. As a result, this may have accelerated the impact of frustration at work on turnover intention through fear of infection among those with shorter working hours under COVID-19 circumstances compared with those with longer working hours, who may have been more accustomed to the circumstances surrounding COVID-19, leading to less worry and familiarity with the situation. The conditional indirect effect was the strongest among those with short working hours. This may be due to the circumstances of the pandemic being unique and thus differing from the normal situation in the healthcare sector.

Additionally, the NHS has been facing a staff shortage, with declining numbers of nurses and general practitioners (GPs), and a lower ratio of doctors per population than the OECD average [9]. This indicates that current HCWs are expected to have a work overload to fill the gap created by staff shortages. A report by the British Medical Association [71] states that routinely working additional hours to make up for staffing deficits has led to dangerously high tiredness and burnout levels among HCWs. Based on current trends and data up to February 2020, which do not include the impacts of COVID-19, the existing workforce gap is projected to double over the next five years [72]. With the above, it can be seen that the underlying cause of turnover intention may be the staff shortage in the NHS. This is consistent with the results of the qualitative interviews; for example, Doctor 14 indicated that the longer working hours and staff shortages are post-pandemic impacts. Further, Doctor 11 mentioned the system being overloaded and that he has to care for multiple patients at the same time, leading to frustration at work.

Our research has uncovered a significant link between frustration at work, fear of infection, working hours, and turnover intention among HCWs in the NHS during the pandemic. These findings make a valuable contribution to the study of turnover intention. As the world recovers from the pandemic, its effect on the healthcare system can be considered long-lasting, with the most notable effect being the relatively high turnover intention among HCWs.

## 7. Recommendations and Limitations

This study’s findings provide insights into the effects of frustration at work and fear of COVID-19 infection and their impacts on turnover intention among HCWs. The results show that the British healthcare workforce struggled to support its doctors in many ways, with pre-existing issues being exacerbated by the global pandemic. In particular, the lack of direction in pandemic policy-making resulted in an overwhelmed system, with the burden falling on the healthcare staff themselves. The UK Government faced widespread criticism for its inadequate response, characterised by errors in decision making, slow enforcement and implementation of lockdowns, the discontinuation of testing, a lack of essential supplies, and insufficient test and trace strategies [6]. Previous studies have shown that resource depletion negatively impacts employees’ commitment to the organisation, leading to a higher turnover intention among workers [73,74]. To moderate the impact of stress and frustration at work, greater organisational support is needed from the NHS. One consideration for greater organisational support would be for hospitals to re-evaluate minimum staffing levels per department. This would allow for a more manageable workload per doctor [75], thus not only improving frustration at work and stress levels but also improve individual patient care [53]. Another way organisations can support trainees is to give readily available psychological support following difficult periods of work or when feeling burnt out [76]. If this support is offered by hospitals and trusts, this shows recognition of the difficult work doctors undertake, and also creates better relationships between employers and employees. Furthermore, fast-tracked support reduces sick leave duration and keeps higher workforce levels overall.

In light of the job being highly stressful, and having a global shortage in doctors, it is important for the Government to keep the job attractive. This can be managed in non-financial ways, including better infrastructure in hospitals. Doctors work long shifts, and some NHS hospitals have poor quality rest areas and a lack of services during unsociable shift hours [77]. Small investments from governments, like 24 h staff access to food and comfortable rest places, can massively improve the quality of time at work with relatively reduced financial investment. These supports from organisations and the Government might help improve HCWs’ quality of life and reduce frustration at work, stress, and turnover intention.

The continuous yearly salary decreases, chronic staff shortages, and unmanageable workloads have culminated in profound job dissatisfaction. This pervasive discontent has had tangible repercussions, with over 15% of NHS doctors taking hard steps towards leaving the service, as noted by Tonkin [78]. From adequate resources to manpower, greater attention needs to be paid to ensuring that HCWs have a reduced stress level and less frustration at work, which could aid in alleviating the high turnover intention. In the years following the pandemic, which has already affected the workforce numbers due to both burnout and sickness, doctors have been striking regarding pay [79]. The UK medical workforce is lower paid than other equal standing countries like Australia. It is to be noted that the UK’s doctor emigration rate is progressively getting higher, with countries like Australia providing better working conditions and salaries, and providing ease with recognising UK trained medical degrees [80]. This is a prominent issue to address to prevent further loss of workforce. The UK could prevent further loss of workforce by providing better working conditions and salaries, which could reduce the degree of staff shortages. Furthermore, it might help alleviate the high levels of stress and frustration at work. Another key point is that the workforce shortage cannot solely be addressed by the UK’s recent push to open more medical schools [81]. There are also training bottlenecks due to a lack of training posts countrywide and across all specialties [82]. This directly prevents doctors from achieving consultant positions, and having their own patient population, as well as exacerbating workforce frustration with inability to progress in their careers. This is demonstrated in the yearly training competition ratios spiralling upwards since 2019 [82]. Clearly, medical school places are not the only concern, as the UK has doctors ready to train but no investment to increase training posts for more consultants. The UK has already taken steps such as putting doctors on the global shortage list as of 2019, which has allowed international medical graduates to apply for doctor roles more easily within the NHS [83]. This also alleviates the shortage, which reduces the HCWs’ stress levels.

Our interviews indicated that staff shortages resulted in a greater work burden on HCWs, which had a rollover impact on their turnover intention. While the government has initiated steps to alleviate the healthcare staffing crisis by increasing medical school admissions, it has not yet successfully integrated these increases into effective training pathways. Amid these systemic challenges, the pandemic has had a direct impact on the professional development of HCWs. During the pandemic, surgical training levels decreased significantly, as all in-person courses, conferences, and exams were cancelled; Health Education England put a hold on scheduled rotations in April 2020 to reduce disruptions [84]. This greatly affected the training process and the fulfilment of specific training goals. With this, the workload on HCWs increased, as they had to take care of multiple patients and even work overtime to meet the demand for health services. Increasing the number of medical school places would have a direct impact on the number of HCWs, thereby reducing the workload of the staff. As indicated by West et al. [85], managing and redistributing job responsibilities are essential to reduce stress on HCWs. Thus, the impact of excessive workload can be reduced by allocating sufficient resources and manpower to the healthcare sector [86]. This can aid in reducing the turnover intention of HCWs, as indicated by research on the job demand–resources model by Demerouti et al. [87] and Bakker et al. [88].

This present study has limitations. Firstly, it is based on the experiences of a limited sample of 119 participants and has the typical shortcomings of a cross-sectional analysis, which prevents testing for causality over time. However, the authors tried to overcome this limitation by using mixed methods to triangulate the findings. Secondly, this study employed an online qualitative survey in which participants responded to open-ended questions on an online platform, Qualtrics. While this approach offers certain advantages, it also has drawbacks compared with traditional face-to-face interviews. For instance, the researcher may have limited the ability to ask follow-up questions. However, the qualitative results were used to supplement the quantitative findings, thereby strengthening the overall conclusions of the study. Thirdly, the sample size for the qualitative survey is relatively small, with only 18 out of 119 participants completing the online self-administered interview. Future research should aim to increase the sample size for qualitative interviews. Another limitation is that the study was conducted during the pandemic, in which HCWs may experience stress, frustration at work, and turnover intention differently from in the post-pandemic period; thus, further study in the post-pandemic period is needed. Future research could examine the factors that influence the turnover intention of HCWs over time in order to determine whether crises alter the importance of certain factors compared with in normal times.

## 8. Conclusions

In conclusion, it was found that the pandemic has greatly affected the daily functioning of the NHS. It introduced a workload that has remained increased, with the workers in the system unable to keep up. COVID-19 exacerbated prior issues, with the key difference being that the HCWs in the system are being driven to their limits. This study shows that the fear of infection derived from COVID-19 and its surrounding circumstances mediated the impact of frustration at work on turnover intention among HCWs in the NHS. Furthermore, working hours moderated the mediation effect of fear of infection on the relationship between frustration at work and turnover intention. This research provides valuable insights into the interplay of frustration at work, fear of infection, working hours, and turnover intention. It is crucial to implement changes to prevent an increase in turnover intention and further exacerbation of understaffing due to skilled workers leaving.

## Figures and Tables

**Table 1:** The UK’s pandemic waves.

Pandemic Wave (Period)	Details of Pandemic Wave
First Wave (Feb.–Sep. 2020)	Initially, the government was ignorant of the seriousness of COVID-19 and deployed a rather laidback strategy to tackle the rising number of infections. Emphasis was put on returning to normal rather than preparing for a potential spike. There were delays in the implementation of public health control measures, which included contact tracing, social distancing measures, and advice to wear face coverings. Often, there were inconsistencies between the advice of HCWs and that which was implemented by the government. With this, the public found it hard to comply with the relevant preventive measures [21]. Further, nearly all HCWs perceived the preparations to control the pandemic situation as inadequate [22].
Second Wave (Sep. 2020–Apr. 2021)	Following a shaky response during the first wave, there were some efforts to introduce social distancing measures across regions from summer 2020. These measures ranged from tiered restrictions to complete lockdowns in order to prevent the spread of disease. Owing to the communication gap between regional and national governments, the social distancing measures introduced were forceless. The confusion seen in the first wave continued into the second, which severely impacted public trust in the government. This was evident from the last-minute changes to the social distancing measures during the Christmas period. Soon after, the vaccine programme commenced in early 2021 [23].
Third Wave (May–Dec. 2021)	In the third wave, the UK witnessed the rapid spread of the Delta variant. Given the mass deployment of vaccines across the UK, the death and hospitalisation rates were lower than those in the previous waves. Despite this, the UK healthcare services were overstretched, as they struggled to provide necessary care to both COVID-19 and non-COVID-19 cases. With this, doctors in private clinics tested patients for COVID-19 before treatment due to a fear of exposure [24]. This demonstrates a change in the norm for clinical treatment, as the healthcare sector looked to reduce the risk of infection for HCWs.
Fourth Wave (Dec. 2021–Jan. 2022)	At this point in the pandemic, the free testing services, self-isolation payments, and statutory sick pay were all stopped by the government. This resulted in a lack of support to the grassroots, as they struggled to protect themselves from the contagious Omicron variant. The lack of government support and guidance resulted in further pressure on the healthcare system. In the midst of this, the uncovering of government officials’ non-observance of the social distancing measures led to further public distrust and the abandonment of such measures by the public. The UK announced that all restrictions introduced to limit the spread of COVID-19 were to end on 26 January 2022 [25].

**Table 2:** Characteristics of respondents.

Characteristics	Frequency	Percent
Gender		
Male	41	34.5
Female	75	63.0
Prefer not to say	3	2.5
Age		
Aged 25 or under	14	11.8
Aged 26 to 35	47	39.5
Aged 36 to 45	27	22.7
Aged 46 to 55	16	13.4
Aged 56 or above	15	12.6
Profession category		
Nurses	26	21.8
Doctors	79	66.4
Therapists	3	2.5
Administration	6	5.0
Pharmacist	3	2.5
Other	2	1.6
Place of work		
Public health sector	113	95.0
Private health sector	5	4.2
Other	1	0.8
Monthly salary		
Less than GBP 1500	4	3.4
GBP 1500–GBP 2999	58	48.7
GBP 3000–GBP 4499	29	24.4
GBP 4500–GBP 5999	10	8.4
More than GBP 6000	12	10.1
Prefer not to say	6	5.0
Working years		
Less than 1 year	14	11.8
1–5 years	50	42.0
6–10 years	23	19.3
11–15 years	14	11.8
More than 15 years	18	15.1
Working schedule		
Fixed working hour	52	43.7
Rotating shifts	45	37.8
Not applicable	22	18.5
Working hours per week		
Less than 32 h	8	6.7
33–40 h	20	16.8
41–48 h	25	21.0
49–56 h	34	28.6
57–64 h	12	10.1
More than 64 h	20	16.8
N	119	100.0

**Table 3:** Mean, standard deviation, and Pearson correlation matrix for continuous variables (N = 119).

	M	SD	1	2	3	4
1. Frustration at work	3.82	1.16				
2. Fear of infection	2.93	1.11	0.33 **			
3. Turnover intention	3.56	1.09	0.24 **	0.30 **		
4. Working hours	3.69	1.48	0.22 *	0.28 **	0.24 **	

Note. * *p* < 0.05; ** *p* < 0.01.

**Table 4:** Mediation of fear of infection between frustration at work and turnover intention (N = 119).

Variables	*B*	SE *B*	β	*R* ^2^	Δ*R*^2^
Step 1				0.33	0.11 ***
Constant	1.71 ***	0.33			
Frustration at work	0.32 **	0.08	0.33		
Step 2				0.34	0.11 ***
Constant	2.26 ***	0.36			
Frustration at work	0.15	0.09	0.16		
Fear of infection	0.25 **	0.09	0.25		

Note. ** *p* < 0.01; *** *p* < 0.001.

**Table 5:** Moderated mediation results (*N* = 119).

Predictors	Fear of Infection				Turnover Intention			
	*B*	SE	*p*	95%CI	*B*	SE	*p*	95%CI
Frustration at work	0.30	0.08	<0.001	[0.139, 0.468]	0.15	0.09	0.09	[−0.022, 0.325]
Frustration at work × working hours	−0.14	0.06	0.011	[−0.256, −0.034]				
Fear of infection					0.25	0.09	0.008	[0.065, 0.427]
*R* ^2^	0.20		<0.001		0.11		<0.001	
*F*	9.74							
Conditional indirect effects	Effect		SE		Boot LLCI		Boot ULCI	
Short working hours	0.13		0.07		0.017		0.287	
Medium working hours	0.07		0.04		0.009		0.172	
Long working hours	0.02		0.03		−0.029		0.097	
	Index		SE		Boot LLCI		Boot ULCI	
Moderated mediation index	−0.04		0.02		−0.089		−0.002	

**Table 6:** The statements of feelings.

Statements
1. I feel more irritable and angrier.
2. I feel sad and depressed.
3. I feel less productive and as though my achievements are not valued.
4. I feel nervous and on edge at work.
5. I am unable to “switch off” after work.
6. I find myself lacking motivation with regards to my career.

## Data Availability

The data presented in this study are not publicly available but are available from the corresponding author upon reasonable request.
